# miR-489-3p promotes malignant progression of non-small cell lung cancer through the inactivation of Wnt/β-catenin signaling pathway via regulating USP48

**DOI:** 10.1186/s12931-022-01988-w

**Published:** 2022-04-12

**Authors:** Pei Zhang, Li Li, Bing Wang, Xu Ran, Shengrong Yang, Yujie Luo, Yunhe Li, Zhenghong Wang, Yi Liu, Bing Zhu

**Affiliations:** Department of Thoracic and Cardiovascular Surgery, The Second Affiliated Hospital of Chongqing Medical University, No.76, Linjiang Road, Yuzhong District, Chongqing, 400010 People’s Republic of China

**Keywords:** NSCLC, miR-489-3p, USP48, Wnt/β-catenin signaling pathway

## Abstract

**Background:**

Non-small cell lung cancer (NSCLC) is the most prevalent form of lung cancer globally, with average age of cancer patients becoming younger gradually. It is of significance to gain a comprehensive understanding of molecular mechanism underlying NSCLC.

**Methods:**

Quantitative polymerase chain reaction (qPCR) and western blot were applied to measure RNA and protein levels separately. Functional assays and western blot were performed to determine the effects of miR-489-3p and USP48 on cell growth, migration and epithelial-mesenchymal transition (EMT) in NSCLC. TOP/FOP flash luciferase reporter assay was carried out to detect the activity of Wnt pathway. Besides, qPCR, RNA pulldown and luciferase reporter assays were conducted to probe into the target gene of miR-489-3p. Immunoprecipitation-western blot (IP-western blot) analysis was implemented to assess the effect of USP48 on the ubiquitination of β-catenin.

**Results:**

miR-489-3p hampers NSCLC cell proliferation, migration and EMT in vitro and NSCLC tumorigenesis and metastasis in vivo. Additionally, miR-489-3p inactivates Wnt/β-catenin signaling pathway and regulates USP48 to inhibit the ubiquitination of β-catenin. Moreover, USP48 propels the development of NSCLC cells.

**Conclusions:**

The current study demonstrated that miR-489-3p promotes the malignant progression of NSCLC cells via targeting USP48, which might offer a new perspective into NSCLC treatment.

**Graphical abstract:**

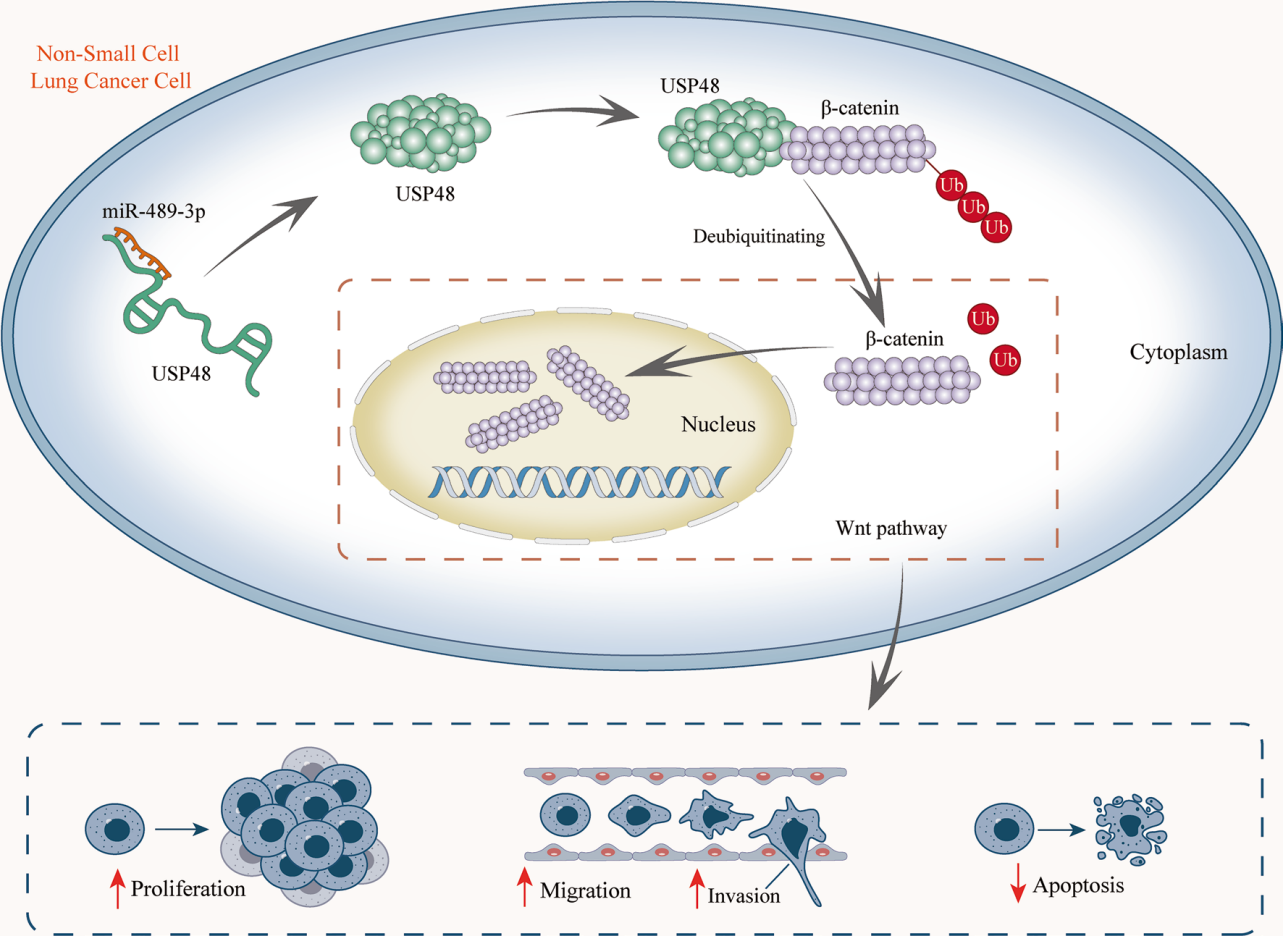

**Supplementary Information:**

The online version contains supplementary material available at 10.1186/s12931-022-01988-w.

## Introduction

As the most prevalent type of lung cancer, NSCLC takes up over 80% of lung cancer cases [1]. In spite of the development of medicine, most NSCLC patients are diagnosed at advanced stage, of whom the five-year survival rate amounted around 15% [2]. The most crucial risk for NSCLC amounts to tobacco consumption, with carcinogenic compounds in tobacco targeting the cells of bronchial mucosa or alveolar epithelium [3, 4]. Immune checkpoint inhibitors (ICIs) have been applied for the treatment of NSCLC patients with the combination of chemotherapy, or have been used to treat stage III patients after definite chemoradiotherapy [5]. However, it is still necessary to identify relevant biomarkers for NSCLC and understand their underlying mechanism.

MicroRNAs (miRNAs) are small non-coding RNAs, featuring the ability of post-transcriptional gene expression regulation via modulating messenger RNA (mRNA). miRNA is a hot topic in NSCLC studies [6, 7]. For instance, miRNA-30 serves as an important regulator in NSCLC [8]; miRNA-621 suppresses the development of NSCLC through regulating SIX4 [9]; serum miRNA-365 targets TTF-1 to serve as a prognostic marker for NSCLC [10]; and overexpressed miR-21 is associated with clinicopathological features and poor prognosis of NSCLC patients [11].

Wnt/β-catenin pathway plays an important role in NSCLC [12]. For example, FOXP3 facilitates the growth and metastasis of tumor in NSCLC via inducing Wnt/β-catenin pathway activation [13]; ZNF671 suppresses the proliferation of NSCLC cells through Wnt/β-catenin pathway [14]; SOX9 propels the capacity of NSCLC cells to migrate and invade via Wnt/β-catenin pathway [15]. As for the mechanism, it has been found that deubiquitination is closely related to Wnt/β-catenin pathway and that many deubiquitinases (DUBs) in the DUB family can reduce the ubiquitination level of β-catenin [16, 17].

Through performing the preliminary experiments, we identified miR-489-3p, which was underexpressed in NSCLC, as the focus of our study. miR-489-3p has been found to regulate the cell progression in melanoma [18], bladder cancer [19], etc. Moreover, it has been reported that MIR503HG inhibits cell proliferation and propels cell apoptosis in NSCLC via modulating miR-489-3p [20]. Although being a key factor in NSCLC, the association of miR-489-3p with NSCLC pathway in NSCLC has never been researched. In our study, we conducted experiments to probe into the regulatory role of miR-489-3p in NSCLC, and its underlying mechanism.

## Materials and methods

### Cell line culture

Human normal lung epithelial cell line (BEAS-2B) and human NSCLC cells (H1299, A549, MIRC5 and SK-LU-1) were commercially attained from ATCC (Manassas, VA, USA). All the cell lines were subjected to cultivation in RPMI 1640 medium (61870127, Gibco, USA), supplemented with 10% fetal bovine serum (FBS, 16000-044, Gibco, USA), 1% penicillin and 1% streptomycin. They were maintained in the incubator with 5% CO_2_ at 37 °C.

### Vector construction and sh-RNA interference

The sequences of USP48 were subcloned into pcDNA3.1 to create overexpression vectors. The sh-RNA targeting USP48 was created by cloning the double-stranded oligos sh-USP48 into the vector pLKO.1. PLKO.1 was commercially acquired from RiboBio (Guangzhou, China). MiR-489-3p agomir and miR-489-3p mimics were used to enhance miR-489-3p for in vitro and in vivo experiments.

### QPCR

Total RNAs were isolated from BEAS-2B, H1299, A549, MIRC5 and SK-LU-1 cells by Trizol (9108, Thermo Fisher, USA). Then, the total RNAs were subjected to reverse transcription and cDNA synthesis through using Reverse Transcription Kit. The RNA samples were measured by ChamQ Universal SYBR qPCR Master Mix (Q711-02, Vazyme, Nanjing, China). The results were calculated and quantified on the basis of 2^−ΔΔCt^ method. Bio-repeats were implemented in triplicate.

### Western blot

Total proteins were subjected to extraction from NSCLC cells by total protein extraction kit. Next, the total proteins were subjected to the treatment with SDS/PAGE gel for separation, followed by being transferred onto the polyvinylidene fluoride (PVDF) membranes. Subsequently, the membranes were subjected to blocking via 5% skim milk, and incubated with the primary antibodies overnight at 4 °C. Next, the membranes were subjected to incubation with the secondary antibodies (ab150077, Abcam, UK). The primary antibodies used contain Anti-E-cadherin (ab40772, Abcam, UK), Anti-Vimentin (ab92547, Abcam, UK), Anti-N-cadherin (ab76011, Abcam, UK), Anti-ZO-1 (ab276131, Abcam, UK), Anti-β-actin (ab8226, Abcam, UK), Anti-Histone H3, Anti-β-catenin (ab32572, Abcam, UK), Anti-c-jun, Anti-c-myc, Anti-GSK-3β, Anti-Axin, Anti-APC and Anti-CK1. Histone H3 and β-actin served as the internal reference. Bio-repeats were implemented in triplicate.

### Chlorhexidine (CHX) treatment

CHX treatment was conducted to assess the degradation of β-catenin in NSCLC cells. The cells were inoculated into 6-well plates and then subjected to USP48 ablation. Afterwards, the transfected cells were treated with CHX. The level of β-catenin was measured by western blot every 4 h till 12 h.

### Cell counting Kit-8 (CCK-8) assay

CCK-8 kit (M4839, ABMOLE, USA) was utilized to perform the experiments. In advance to the experiments, the transfected NSCLC cells were inoculated into 96-well culture plates. Subsequently, the transfected cells were subjected to incubation for 24, 48 and 72 h, followed by CCK-8 solution treatment. Microplate reader (51119770DP, Thermo Fisher) was applied for the calculation of the absorbance value at 450 nm.

### Transwell assay

Migratory capacity of H1299 and A549 cells was evaluated by use of transwell chambers without Matrigel. Transfected cells and serum-free medium were placed in the upper chambers, while complete medium in the lower chambers. After 24 h, the upper chambers were abraded with cotton swab. The cells migrating to the lower chambers were treated with 4% paraformaldehyde (PFA) for fixation, and 0.5% crystal violet for staining. The images of migrated cells were captured using the microscope.

### Terminal deoxynucleotidyl transferase dUTP nick end-labeling (TUNEL) assay

The transfected H1299 and A549 cells were subjected to fixation in 4% PFA at room temperature for a quarter. TUNEL staining was carried out. DAPI was adopted for the staining of the nuclei. Fluorescence microscope was applied to count TUNEL-positive cells. The ratio of TUNEL-positive cells to total cell count was for the evaluation of cell apoptosis.

TUNEL assay was also implemented to assess the apoptosis rate of paraffin embedded tumors slides in each group. All the slides were subjected to coloration of TUNEL staining for the assessment of apoptosis. Matlab was applied to quantify the degree of apoptosis by analyzing the brown area dyed by TUNEL.

### Wound healing assay

Wound healing assay was implemented for the assessment of cell migratory capacity. Transfected NSCLC cells were subjected to the inoculation in 6-well plates for the assay. After the cells grew to 90–95% confluences, the wounds were scratched by use of the pipette tip. 24 h after the scratch, the images of wound were obtained.

### RNA pulldown assay

The biotinylated wild-type (Wt) USP48 3′ untranslated region (UTR) and biotinylated mutated (Mut) USP48 3′ UTR were fostered. Afterwards, structure buffer was mixed with bio-labeled RNAs for the formation of secondary structure. Next, bio-labeled RNAs were subjected to heating and ice bath for denaturing, and then denatured and bio-labeled RNAs were incubated with Pierce™ Streptavidin Magnetic Beads (88816, Thermo Fisher, USA). Afterwards, H1299 and A549 cells were lysed, and lysates were subjected to the incubation with magnetic beads for 4 h at 4 °C. After the incubation, the beads were eluted, and the pulldown products were treated with Trizol for extraction of miR-489-3p. QPCR was implemented to detect the enrichment of miR-489-3p.

### In vivo xenograft model

Male BALB/c nude mice aging 6–8 weeks were commercially obtained from Shanghai Lab. Animal Research Center. Nude mice were divided into four random groups (n = 3–5). 48 h after the transfection of miR-489-3p agomir and agomir NC into A549 cells, 100 μL of 2 × 10^7^ transfected cells were obtained for the animal experiments. 2 × 10^6^ cells were subjected to subcutaneous injection into each nude mouse. 7 days later, the tumor formation was accomplished. The volume of tumors was measured every 7 day. 28 days after the injection, nude mice were sacrificed for the weighing of tumors.

To evaluate tumor metastasis, animal experiments were performed using A549 cells. A549 cells were subjected to the transfection with miR-489-3p agomir and agomir NC. After 48 h, the cells were made into suspension and mixed with Matrigel 1:1 to prepare for cell injection. 100 μL of 2 × 10^7^ transfected cells were obtained for the animal experiments. 2 × 10^6^ cells were subjected to the injection into each nude mouse. Tail vein injection was applied to simulate the pathway of tumor metastasis to liver in situ. After 6 weeks, the livers of nude mice were taken out for routine sections. After HE staining, the livers were observed under microscope. The number of metastatic colonies was calculated. The whole experiments were approved by ethic committee of the Second Affiliated Hospital of Chongqing Medical University.

### Immunohistochemical (IHC) analysis

Ki67 antibodies and proliferating cell nuclear antigen (PCNA) antibodies were used for IHC. Tumor tissues of nude mice were subjected to the treatment with hydrogen peroxide, followed by being incubated with primary antibodies against Ki67 and PCNA overnight at 4 °C. Afterwards, the secondary antibodies (ab150077, Abcam, UK) were incubated with tissues at 37 °C. After 1 h, tissues were dyed by DAB. The expression of Ki67 or PCNA was defined by IHC score.

### Colony formation assay

Transfected H1299 and A549 cells were cultured in 6-well plates (400/well). The plates were subjected to the incubation at 37 °C with 5% CO_2_ for 14 days. 14 days later, cell colonies were subjected to the staining using crystal violet solution and counted under the microscope.

### Coimmunoprecipitation (Co-IP)

Co-IP was conducted for the validation of the interaction between β-catenin and USP48. NSCLC cells were lysed by IP lysis buffer. The lysate was subjected to incubation with the primary antibodies against USP48, β-catenin (ab32572, Abcam, UK) and IgG (ab133470, Abcam, UK). After being washed in IP lysis buffer, cell samples were prepared for western blot analysis. Three independent bio-repeats were conducted in the experiment and each bio-repeat contains three technical replicates.

### Luciferase reporter assay

Dual Luciferase Reporter Gene Assay Kit (RG027, Beyotime, Shanghai, China) was employed to implement luciferase reporter assay. USP48 3’UTR-Wt and USP48 3’UTR-Mut were subcloned into the pmirGLO vector. Next, miR-489-3p mimics and mimics NC were co-transfected with luciferase reporter vectors. 48 h after transfection, luciferase activity was detected relative to Renilla luciferase activity. To detect the activity of signaling, we employed Notch signaling pathway kit (GK025, Sciencell, USA), Wnt signaling pathway kit (GK026, Sciencell, USA), PI3K/Akt signaling pathway kit (K-1000, J&K Scientific, Wuhan, China), Nanog signaling pathway kit (ab236720, Abcam, UK), Hippo-YAP signaling pathway kit (YSK8132, Y-S Biotechnology, Shanghai, China), Hedgehog signaling pathway kit (CSB-YP729058PYX, CUSABIO, Wuhan, China) and NF-κB signaling pathway kit (ZN-SP712, Hefei Zhi En Biology, Hefei, China).

### TOP/FOP flash luciferase reporter assay

The assay was implemented to detect the signal activity of Wnt signaling pathway. The TOP Flash/FOP Flash reporter vectors were adopted for the co-transfection with mimics NC or miR-489-3p mimics into H1299 and A549 cells. 48 h later, TOP/FOP luciferase activities were detected.

### Statistical analysis

The data in all the experiments were presented as the mean ± SD. Student’s t-test was used for difference comparison between two groups, while one-way or two-way analysis of variance (ANOVA) for three groups or more. P value < 0.05 was considered to be statistical significant.

## Results

### miR-489-3p attenuates the malignant progression of NSCLC in vitro

**Fig. 1:**
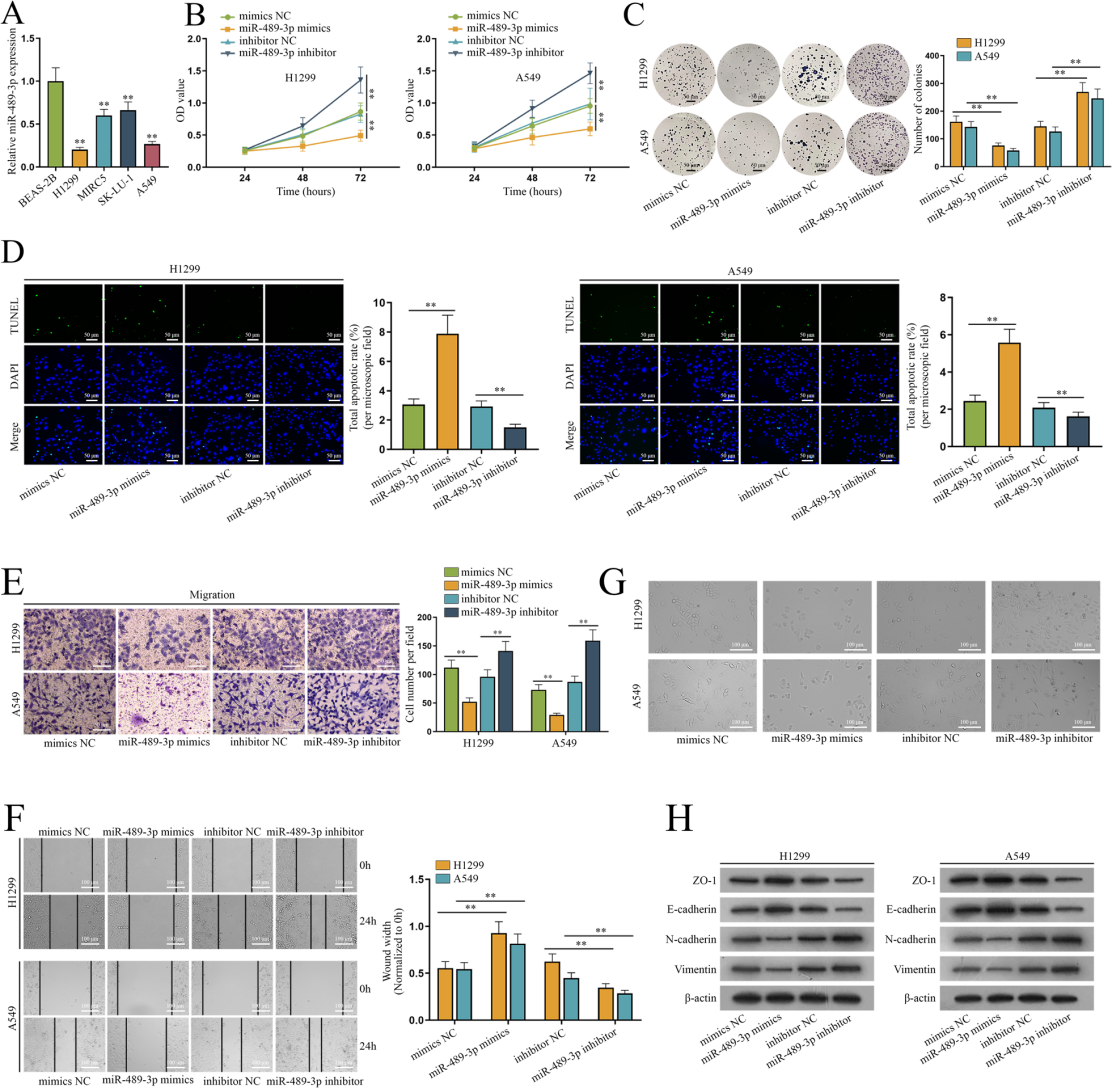
MiR-489-3p inhibits the malignant progression of NSCLC in vitro. **A** QPCR was conducted to assess miR-489-3p expression in BEAS-2B, H1299, A549, MIRC5 and SK-LU-1 cell lines. **B** and **C** CCK-8 and colony formation assays were conducted to evaluate the proliferation of H1299 and A549 cells after the inhibition or overexpression of miR-489-3p. **D** TUNEL assay was performed to assess the effects of the ectopic expression of miR-489-3p on cell apoptosis in NSCLC. **E** and **F** Transwell and wound healing assays were performed to evaluate the migratory capacity of H1299 and A549 cells after the inhibition or overexpression of miR-489-3p. **G** Microscope was employed to observe the shapes of H1299 and A549 cells after the inhibition or overexpression of miR-489-3p. **H** Western blot evaluated the protein levels of ZO-1, E-cadherin, N-cadherin and Vimentin after the inhibition or overexpression of miR-489-3p. **P < 0.01

Firstly, we used GEPIA2 (http://gepia2.cancer-pku.cn/#index) to evaluate the association between miR-489-3p expression and survival rate of lung squamous cell carcinoma (LUSC) patients. LUSC is the main subtype of NSCLC. As indicated in Additional file 2, low-expressed miR-489-3p leads to poor prognosis of LUSC patients. Afterwards, we detected miR-489-3p expression in NSCLC cell lines (H1299, MIRC5, SK-LU-1 and A549) and normal cell line BEAS-2B. It’s unearthed by qPCR analysis that miR-489-3p is markedly expressed at low levels in NSCLC cell lines compared to BEAS-2B cell line. Particularly, H1299 and A549 cells display a relatively lower expression of miR-489-3p (Fig. 1A). Thereby, we chose H1299 and A549 cells for the follow-up investigations. Additionally, the overexpression efficiency of miR-489-3p mimics and interference efficiency of miR-489-3p inhibitor in H1299 and A549 cells were elucidated via qPCR (Additional file 1: Fig. S1A). Next, the functional assays were implemented to evaluate the biological role of miR-489-3p in NSCLC. CCK-8 and colony formation assays were implemented for the assessment of cell proliferation in NSCLC. As indicated in CCK-8 assay, it’s shown that the transfection of miR-489-3p mimics led to a drop in absorbance in H1299 and A549 cells while miR-489-3p inhibitor caused an increase in absorbance (Fig. 1B). Likewise, the number of colonies was significantly decreased in miR-489-3p mimics groups relative to controls but increased in miR-489-3p inhibitor groups compared to controls (Fig. 1C). The above results indicated that miR-489-3p suppresses NSCLC cell growth in vitro. In addition, TUNEL assay evaluated cell apoptosis. It’s shown that miR-489-3p overexpression caused an increase in apoptosis rate while miR-489-3p inhibition caused a drop in apoptosis rate compared to controls (Fig. 1D), indicating that miR-489-3p induces the apoptosis of NSCLC cells. Subsequently, we implemented transwell and wound healing assays to evaluate the migratory capacity of NSCLC cells after the upregulation or downregulation of miR-489-3p expression. As shown in Fig. 1E, migrated cells were decreased after the enhancement of miR-489-3p but increased after the inhibition of miR-489-3p, indicating that miR-489-3p represses cell migration in NSCLC. Wound assays further verified this finding as wound widths after the upregulation of miR-489-3p were broader than controls but narrower than control after the downregulation of miR-489-3p (Fig. 1F). Subsequently, we observed phenotypic changes and measured expressions of EMT markers to assess the effects of miR-489-3p on EMT in NSCLC. As shown in Fig. 1G, the number of spindle-shaped cells in miR-489-3p mimics groups was obviously less than controls but that in miR-489 inhibitor groups was more than controls. Then, we detected the EMT-associated proteins including ZO-1, E-cadherin, N-cadherin and Vimentin, among which the former two are negatively related to EMT while the latter two positively-related. It’s unearthed by western blot that overexpressed miR-489-3p improved the protein level of ZO-1 and E-cadherin but diminished that of N-cadherin and Vimentin in H1299 and A549 cells; at the same time, silenced miR-489-3p had the opposite effects on the EMT-related proteins (Fig. 1H). Altogether, miR-489-3p impairs NSCLC cell proliferation, migration, EMT but propels cell apoptosis in vitro.

### miR-489-3p hampers NSCLC tumorigenesis and metastasis in vivo

**Fig. 2:**
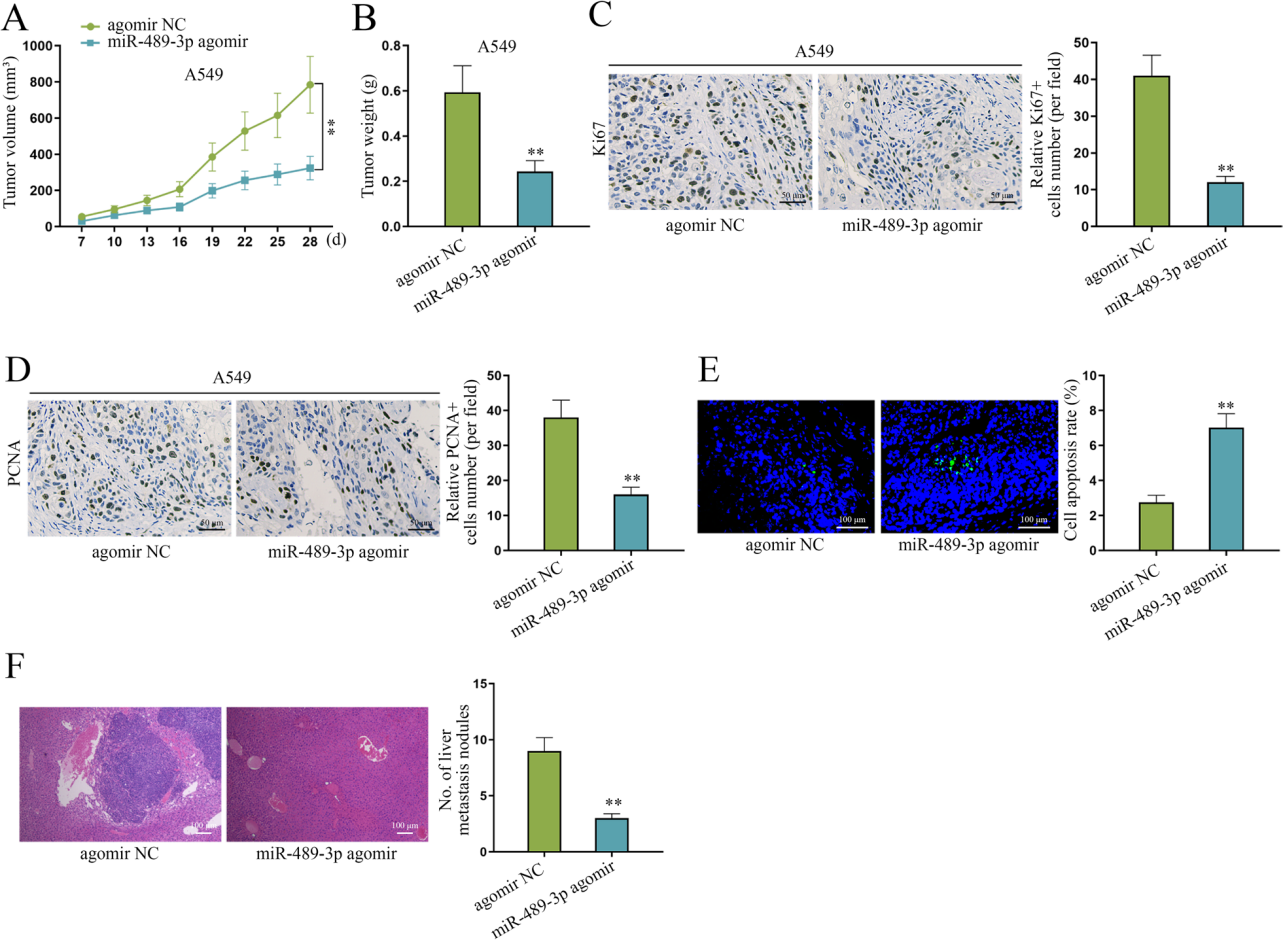
MiR-489-3p hampers NSCLC tumorigenesis and metastasis in vivo. **A**–**B** Animal experiments were conducted to evaluate the effect of miR-489-3p on NSCLC tumor growth. **C**–**D** IHC assays detected the expression of Ki67 and PCNA in NSCLC tumor tissues. **E** TUNEL assays were used to evaluate the apoptosis rate of NSCLC tumor tissues. **F** In vivo metastatic experiments were used to evaluate the effect of miR-489-3p on metastasis of NSCLC tumors. **P < 0.01

Besides in vitro functional assays, we conducted in vivo experiments for the exploration of the effects of miR-489-3p on NSCLC tumorigenesis. The results showed that volume of tumors excised from the mice injected with miR-489-3p agomir-transfected A549 cells grew slower than that of control (Fig. 2A). In addition, the tumors in miR-489-3p agomir groups were clearly smaller and lighter than those in control groups (Fig. 2B). Furthermore, we conducted IHC to detect proliferation markers (PCNA and Ki-67) in tissues dissected from xenografted tumors. The results showed that PCNA- and Ki-67-positive cells in miR-489-3p agomir groups were both less than those in controls (Fig. 2C, D). Also, we performed TUNEL assay in the paraffin-embedded section of tissues dissected from xenografted tumors. The results showed that miR-489-3p agomir groups display a higher apoptosis rate relative to controls (Fig. 2E). Collectively, the above results indicated that miR-489-3p inhibits NSCLC tumorigenesis in vivo. Moreover, in vivo metastatic experiments showed that liver metastasis nodules were obviously reduced in miR-489-3p agomir groups relative to controls (Fig. 2F), which indicated that miR-489-3p hampers tumor metastasis in vivo. Taken together, miR-489-3p impedes NSCLC tumorigenesis and tumor metastasis in vivo.

### miR-489-3p inactivates Wnt/β-catenin signaling pathway

**Fig. 3:**
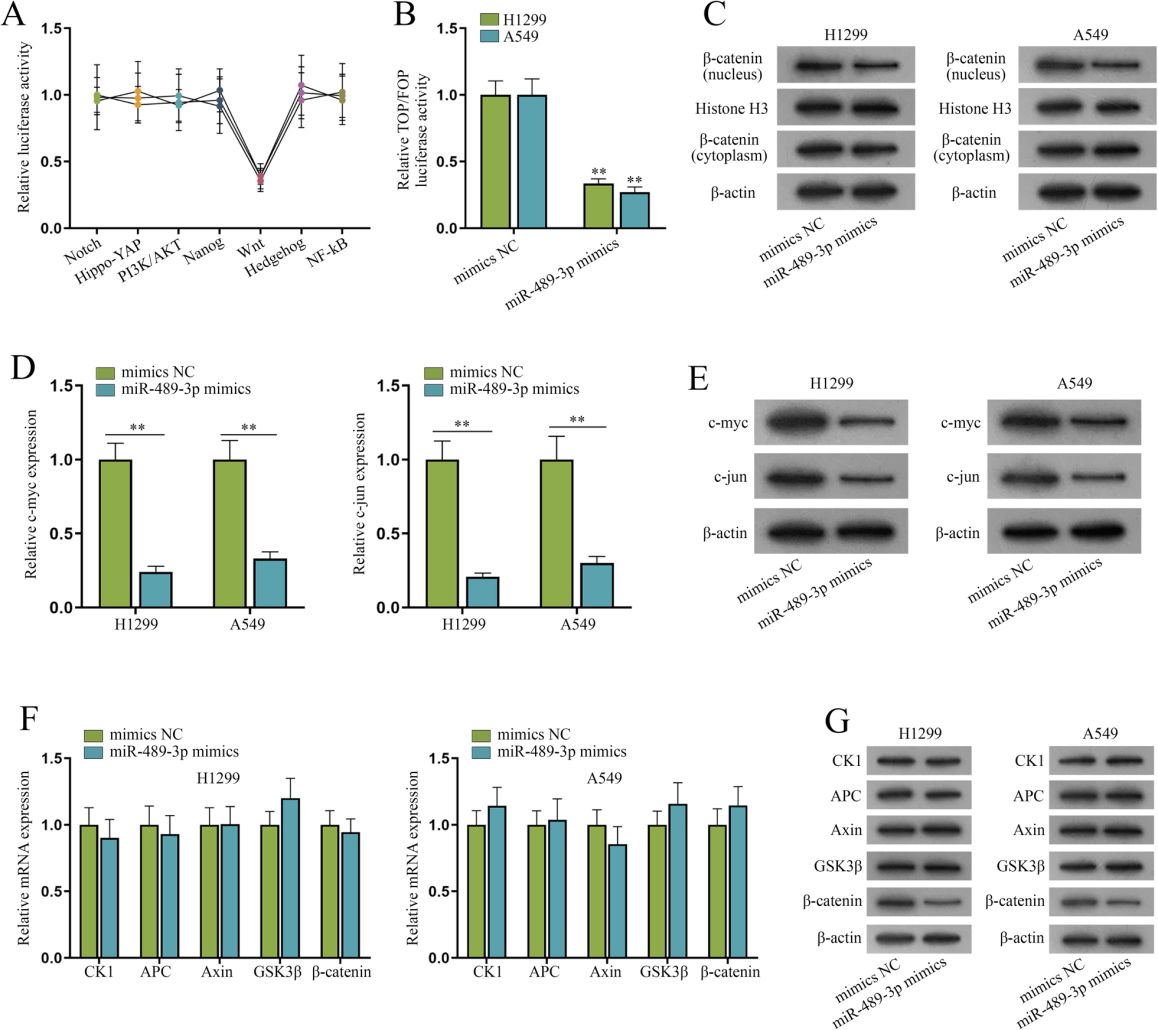
MiR-489-3p inactivates Wnt/β-catenin signaling pathway. **A** Signaling pathway kits were used to assess the influence of miR-489-3p on Notch, Wnt, PI3K/Akt, Nanog, Hippo-YAP, Hedgehog and NF-κB signaling pathways. **B** TOP/FOP flash luciferase reporter assay evaluated the signal activity of Wnt pathway after the enhancement of miR-489-3p. **C** β-catenin expression in the cytoplasm and nucleus of H1299 and A549 cells was testified by western blot after the overexpression of miR-489-3p. **D**–**E** Western blot and qPCR were performed to testify the levels of c-myc and c-jun in H1299 and A549 cells after the overexpression of miR-489-3p. **F**–**G** Western blot and qPCR assessed the levels of β-catenin, CK1, APC, Axin and GSK3β in H1299 and A549 cells after the overexpression of miR-489-3p. **P < 0.01

Given multiple signaling pathways exert crucial functions in NSCLC [12], we conjectured that miR-489-3p might modulate the activation of signaling pathways to inhibit NSCLC. Several pathway reporter kits were utilized to detect whether miR-489-3p affects the activity of signaling pathways in H1299 and A549 cells. It’s shown that the luciferase activity of Wnt pathway was markedly weakened after the transfection of miR-489-3p (Fig. 3A). The TOP/FOP flash reporter assays further verified the inhibitory effect of miR-489-3p on the activity of Wnt pathway as miR-489-3p mimics largely inhibits the TOP/FOP flash luciferase activity of Wnt pathway (Fig. 3B). The activation of Wnt pathway causes an increase in the expression of nuclear β-catenin but a decrease in that of cytoplasmic one [21]. Thereby, we conducted western blot analysis of cytoplasmic and nuclear β-catenin in H1299 and A549 cells. The results showed a drop in nuclear β-catenin in miR-489-3p mimics-transfected NSCLC cells (Fig. 3C), which also verified that miR-489-3p suppresses Wnt pathway in NSCLC cells. Furthermore, we applied qPCR and western blot to analyze the effects of miR-489-3p overexpression on the downstream targets of Wnt pathway (c-myc and c-jun). The results showed that miR-489-3p overexpression diminished the mRNA and protein levels of c-myc and c-jun (Fig. 3D, E), verifying that miR-489-3p could modulate Wnt pathway. Next, we conducted qPCR and western blot to evaluate whether miR-489-3p affects the expression of pivotal proteins of Wnt pathway, including CK1, APC, Axin, GSK3β and β-catenin. It was unmasked that only β-catenin protein expression was reduced while β-catenin mRNA and others had no marked change after the overexpression of miR-489-3p (Fig. 3F, G). To sum up, miR-489-3p inhibits Wnt/β-catenin pathway via regulating β-catenin protein in NSCLC cells.

### miR-489-3p mediates USP48 to inhibit the ubiquitination of β-catenin

**Fig. 4:**
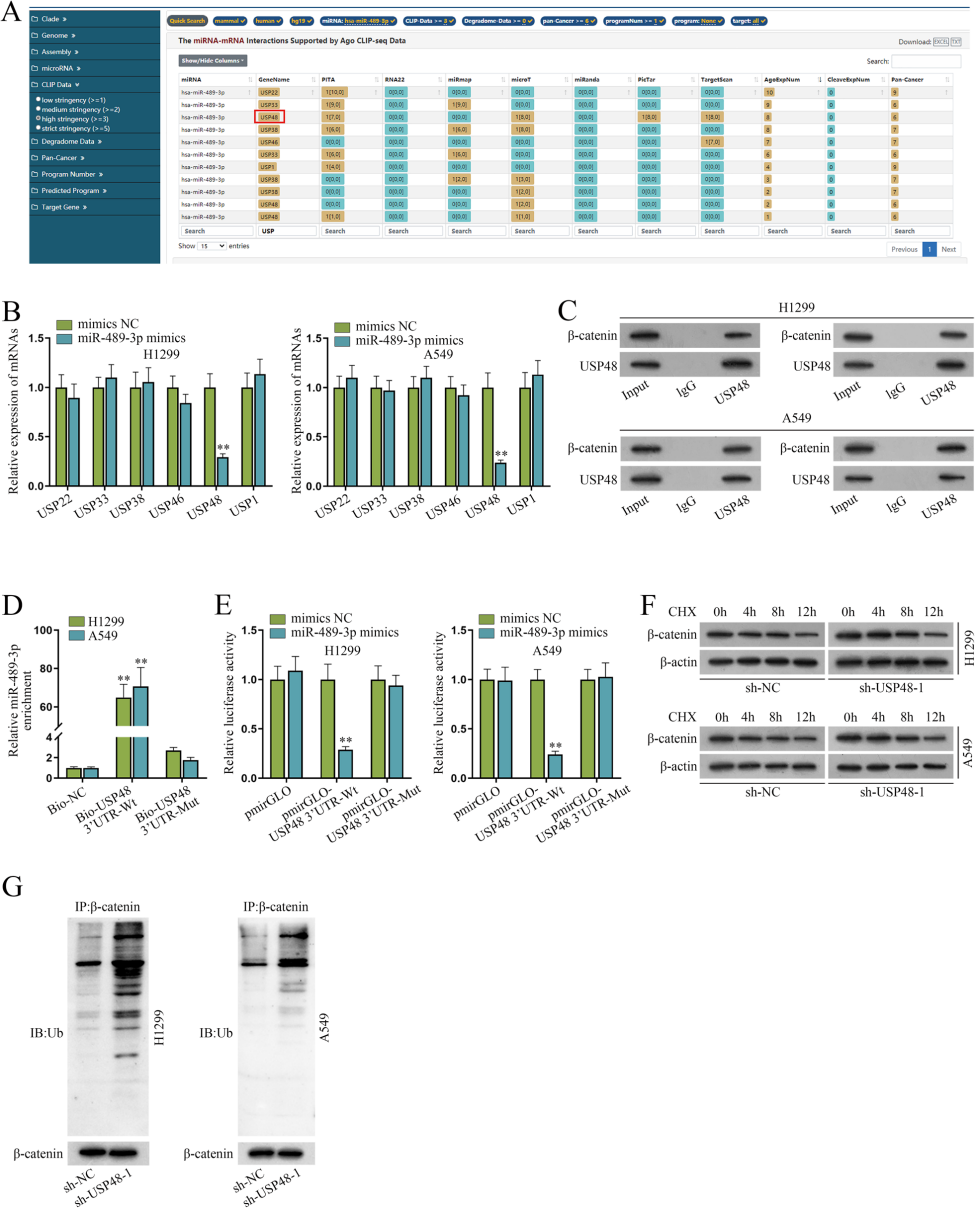
MiR-489-3p mediates USP48 to inhibit the ubiquitination of β-catenin. **A**–**B** The starBase and qPCR screened out USP48 as the potential downstream target of miR-489-3p. **C** Co-IP assays proved the interaction between β-catenin and USP48. **D**–**E** RNA pulldown and luciferase reporter assays were performed for the detection of the interaction between USP48 and miR-489-3p. **F** Western blot detected the protein levels of β-catenin after CHX treatment and USP48 inhibition. **G** IP-western blot detected the ubiquitination of β-catenin after USP48 inhibition. **P < 0.01

Next, we further investigated the regulatory mechanism of miR-489-3p on the expression of β-catenin protein in NSCLC cells. As reported in previous studies, deubiquitination is correlated with Wnt/β-catenin pathway and deubiquitinating enzymes (DUBs) induce the ubiquitination of β-catenin [16]. Therefore, we probed into the downstream DUBs of miR-489-3p. 6 DUBs were predicted by the bioinformatics starBase, including USP22, USP33, USP38, USP46, USP48 and USP1 (Fig. 4A). Then we applied qPCR for the identification of the most relevant DUB of miR-489-3p in NSCLC. The results showed that in NSCLC cells, only the level of USP48 was overtly reduced while other candidate DUBs had no marked change after miR-489-3p enhancement (Fig. 4B), suggesting that USP48 might be the target gene of miR-489-3p in NSCLC. Afterwards, Co-IP assay was performed to detect the interaction between USP48 and β-catenin proteins. The results showed that USP48 was co-immunoprecipitated with β-catenin (Fig. 4C), verifying the interaction between them. Subsequently, it was unearthed by the results of RNA pulldown assays that miR-489-3p was highly enriched in the complex pulled down by Bio-USP48 3’UTR-Wt instead of Bio-USP48 3′ UTR-Mut (Fig. 4D), indicating that miR-489-3p binds to USP48 in NSCLC cells. As shown in Fig. 4E, the luciferase activity of pmirGLO-USP48 3′ UTR-Wt was dramatically weakened by miR-489-3p mimics compared to controls while that of pmirGLO-USP48 3’UTR-Mut displayed no marked change relative to that of pmirGLO-USP48 3′ UTR-Wt (Fig. 4E), indicating that miR-489-3p binds with USP48 3’UTR rather than USP48 3′ UTR-Mut. We used qPCR to assess the efficiency of sh-USP48-1/2/3 and pcDNA3.1-USP48. Due to the higher efficiency, sh-USP48-1 and sh-USP48-2 were selected for the follow-up assays (Additional file 1: Fig. S1B). Given that USP48 is a DUB and some of DUB family members can lower the ubiquitination of β-catenin [16, 17], we further investigated whether USP48 downregulates the ubiquitination of β-catenin in NSCLC cells. Western blot was applied for expression analysis of β-catenin after CHX (a protein synthesis inhibitor) treatment. The results showed that after CHX treatment, β-catenin protein level was diminished at a slower rate after the knockdown of USP48 (Fig. 4F), indicating that USP48 modulates the protein level of β-catenin via affecting its protein degradation instead of protein synthesis. Through IP-western blot analysis, ubiquitination of β-catenin was enhanced after the ablation of USP48 (Fig. 4G), indicating that USP48 deubiquitinates β-catenin. In conclusion, miR-489-3p modulates USP48 to inhibit the ubiquitination of β-catenin in NSCLC cells.

### miR-489-3p propels the malignant phenotypes of NSCLC cells via USP48

**Fig. 5:**
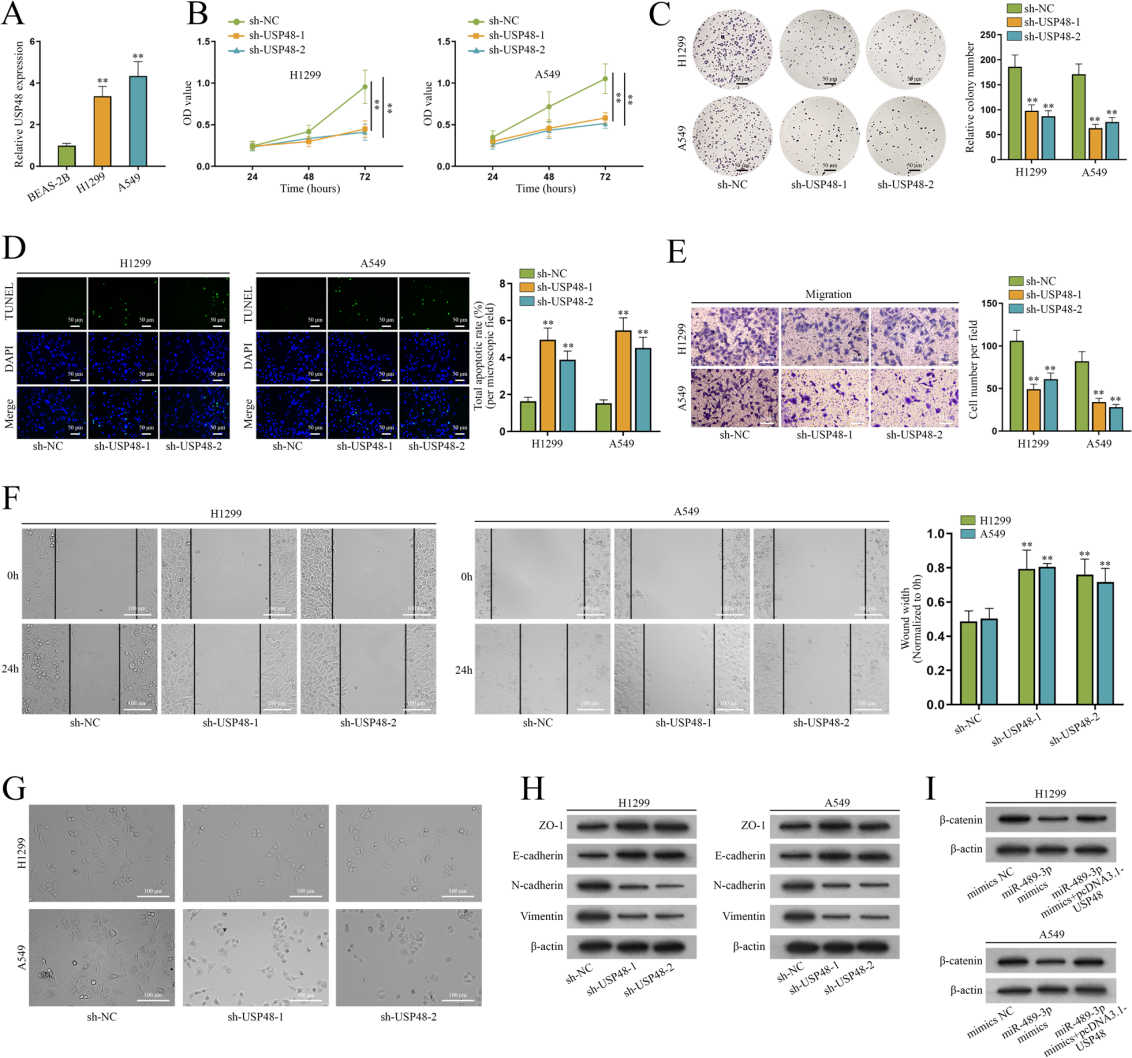
MiR-489-3p propels the malignant phenotypes of NSCLC cells via USP48. **A** QPCR assessed the expression of USP48 in BEAS-2B, H1299 and A549 cells. **B**–**C** CCK-8 and colony formation assays were implemented to evaluate the proliferation of H1299 and A549 cells after the inhibition of USP48. **D** TUNEL assays were performed to assess cell apoptosis after the silencing of USP48. **E**–**F** Evaluation of H1299 and A549 cell migration was assessed by transwell and wound healing assays after USP48 ablation. **G** Microscope was employed to observe the shapes of H1299 and A549 cells after USP48 ablation. **H** Western blot evaluated the protein levels of ZO-1, E-cadherin, N-cadherin and Vimentin after USP48 interference. **I** Western blot assessed the levels of β-catenin after the transfection of mimics NC, miR-489-3p mimics or miR-489-3p mimics + pcDNA3.1-USP48. **P < 0.01

Next, we investigated the role USP48 plays in the cellular progression of NSCLC. Based on the qPCR analysis, we discovered that USP48 was expressed at high levels in NSCLC cell lines (H1299 and A549) relative to that in BEAS-2B cell line (Fig. 5A). It’s unmasked by CCK-8 and colony formation assays that the ablation of USP48 caused the decrease in absorbance and the number of colonies (Fig. 5B, C), which indicated that USP48 knockdown inhibits cell growth in NSCLC. According to TUNEL assay, USP48 knockdown obviously improved the apoptosis rate (Fig. 5D), which indicated that USP48 inhibits cell apoptosis in NSCLC. Transwell and wound healing assays showed that migrated cells were reduced and wound widths were wider relative to controls after the transfection of sh-USP48-1/2 (Fig. 5E, F), which indicates that migratory capacity of NSCLC cells were inhibited by depletion of USP48. Additionally, spindle-shaped cells were clearly reduced due to the downregulation of USP48 (Fig. 5G); the protein levels of ZO-1 and E-cadherin were enhanced while those of N-cadherin and Vimentin were attenuated after the silencing of USP48 in NSCLC cells (Fig. 5H). The above results indicated that silenced USP48 inhibits the EMT of NSCLC cells. As shown in the rescue experiments, USP48 overexpression could reserve the inhibitory effect of miR-489-3p mimics on β-catenin protein in NSCLC cells (Fig. 5I). To sum up, miR-489-3p stimulates NSCLC cell growth, migration and EMT but represses cell apoptosis via regulating USP48.

## Discussion

NSCLC is one of the most common subtypes of lung cancer, threatening the health of population across the globe. Although the combination of ICIs with chemotherapy have been applied to treat NSCLC patients [5], it’s still of necessity to identify biomarkers for NSCLC treatment.

MiRNAs have been found to participate in the regulation of NSCLC. MiRNA-30 [8], miRNA-621 [9], miRNA-365 [10] and miR-21 [11] were reported to regulate NSCLC. In our study, it’s found that miR-489-3p was underexpressed in NSCLC cell lines. Previously, miR-489-3p has been reported to suppress cell proliferation and migration of bladder cancer via downregulating histone deacetylase 2 [19]. In addition, miR-489-3p inhibits proliferation, migration, and invasion in melanoma cells via silencing SIX1 [18]. In line with these findings, miR-489-3p serves as a suppressor in NSCLC cells. Functional experiments, cell shape observation and western blot proved that miR-489-3p suppresses the proliferation, migration and EMT but enhances the apoptosis of NSCLC cells. For further verification, in vivo experiments, TUNEL assays and IHC assays proved that miR-489-3p inhibits the formation and metastasis of NSCLC tumors. However, the correlation of miR-489-3p with signaling pathways in NSCLC has never been researched. Next, the relationship between Wnt/β-catenin pathway and miR-489-3p in NSCLC cells was probed.

The important role of Wnt/β-catenin pathway in NSCLC was validated [12]. Our study conducted experiments to explore underlying mechanism of Wnt/β-catenin pathway in NSCLC cells. Luciferase reporter assays were performed to prove the effect of miR-489-3p on this pathway. Western blot was then conducted to evaluate the translocation of β-catenin in NSCLC cells, verifying that the pathway is inhibited by miR-489-3p. Afterwards, we performed western blot and qPCR, and found out that miR-489-3p influenced downstream target proteins of the pathway; furthermore, related proteins of the pathway were affected by miR-489-3p at protein level, instead of mRNA level.

Deubiquitination is closely associated with Wnt/β-catenin signaling pathway and many DUBs from the DUB family negatively modulate the ubiquitination level of β-catenin [16, 17]. Among the members of DUB family, USP48 has been reported to regulate glioblastoma [22], lung cancer [23], as well as head and neck squamous carcinoma [24]. In our study, we used the starBase and qPCR to screen out USP48. Afterwards, Co-IP, RNA pulldown, and luciferase reporter assays verified the interaction of USP48 with β-catenin. Through CHX treatment, we proved that USP48 regulates β-catenin via its degradation in NSCLC cells. IP-western blot was conducted to prove that USP48 reduces ubiquitination level of β-catenin in NSCLC cells. Subsequently, functional experiments, cell shape observation and western blot proved that inhibited USP48 suppresses the proliferative and migratory capacities and EMT but promotes the apoptosis of NSCLC cells. Rescue experiments demonstrated that miR-489-3p regulates growth, migration, EMT as well as apoptosis in NSCLC cells via USP48.

## Conclusions

In conclusion, miR-489-3p promotes malignant progression of NSCLC cells by regulating USP48 for the inactivation of Wnt/β-catenin pathway. miR-489-3p might be a potential therapeutic target for NSCLC, and the findings about it contribute to the targeted therapies of NSCLC. Compared with the previous studies, our study firstly proved that miR-489-3p suppresses the malignant progression of NSCLC cells, and USP48 propels the malignant progression of NSCLC cells. In addition, it has firstly been proved that miR-489-3p can regulate USP48 to reduce ubiquitination of β-catenin, thus inactivating Wnt/β-catenin pathway in NSCLC cells. However, lack of clinicopathological analysis undermines the stringency of our study. In addition, sequencing and microarray should be utilized to evident that miR-489-3p is a key factor in NSCLC. In the future, we will adopt sequencing and microarray and provide more clinicopathological relevance in our further studies.

## Supplementary Information

**Additional file 1: Figure S1.** The efficiency of plasmids concerning USP48 and miR-489-3p was detected. (A) The efficiency of miR-489-3p mimics and miR-489-3p inhibitor was detected by qPCR in H1299 and A549 cells. (B) The efficiency of sh-USP48-1/2/3 and pcDNA3.1/USP48 was assessed by qPCR in H1299 and A549 cells. **P<0.01.

**Additional file 2:** Overall Survival correlated with expression of miR−489−3p in TCGA−LUSC.

## Data Availability

Not applicable.

## Abbreviations

NSCLC: Non-small cell lung cancer
qPCR: Quantitative polymerase chain reaction
EMT: Epithelial–mesenchymal transition
IP: Immunoprecipitation
ICIs: Immune checkpoint inhibitors
miRNAs: MicroRNAs
mRNA: Messenger RNA
DUBs: Deubiquitinases
PVDF: Polyvinylidene fluoride
CHX: Chlorhexidine
CCK-8: Cell counting Kit-8
PFA: Paraformaldehyde
TUNEL: Terminal deoxynucleotidyl transferase dUTP nick end-labeling
Wt: Wild-type
3′ UTR: 3′ Untranslated region
IHC: Immunohistochemical
PCNA: Proliferating cell nuclear antigen
Co-IP: Coimmunoprecipitation
ANOVA: Analysis of variance
