# Supplementary figures and images for: miR-489-3p promotes malignant progression of non-small cell lung cancer through the inactivation of Wnt/β-catenin signaling pathway via regulating USP48
Source: Respir Res. 2022 Apr 12;23:93. doi: 10.1186/s12931-022-01988-w (PMC9006470; doi:10.1186/s12931-022-01988-w)

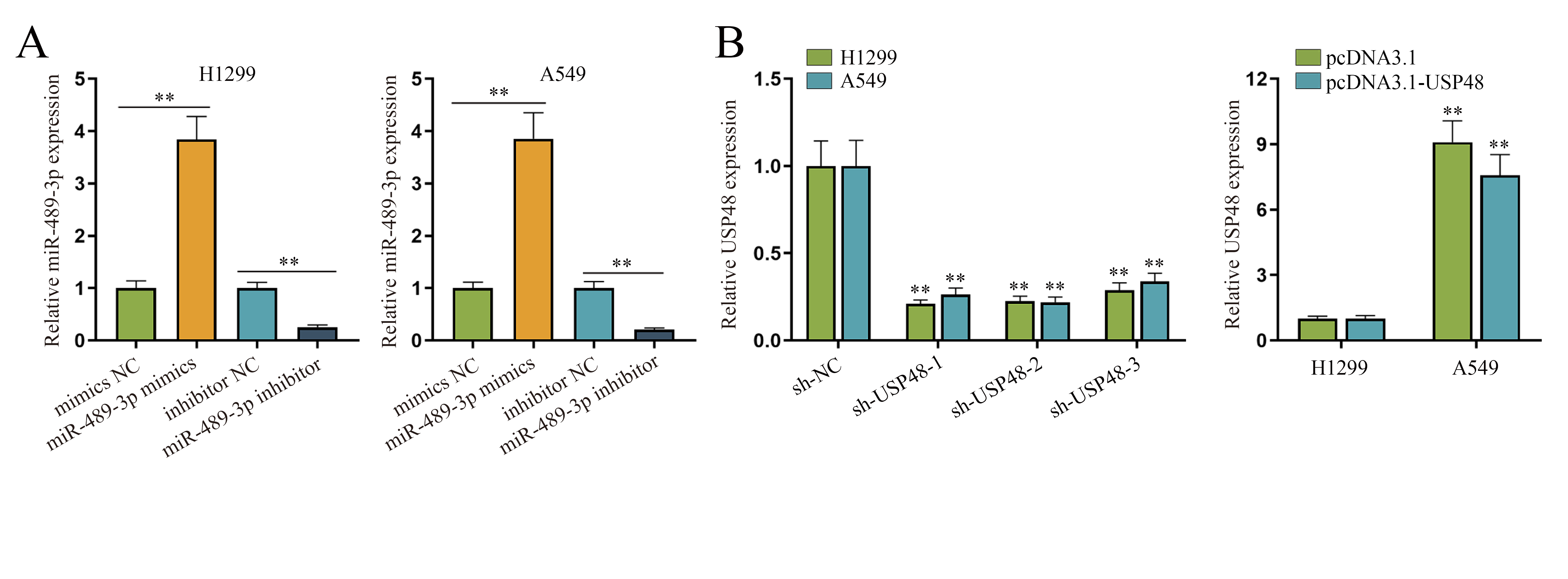

Supplement: Supplementary file 1 — Additional file 1: Figure S1. The efficiency of plasmids concerning USP48 and miR-489-3p was detected. (A) The efficiency of miR-489-3p mimics and miR-489-3p inhibitor was detected by qPCR in H1299 and A549 cells. (B) The efficiency of sh-USP48-1/2/3 and pcDNA3.1/USP48 was assessed by qPCR in H1299 and A549 cells. **P<0.01. [file 12931_2022_1988_MOESM1_ESM.tif]

# Overall Survival correlated with expression of miR-489-3p in TCGA-LUSC

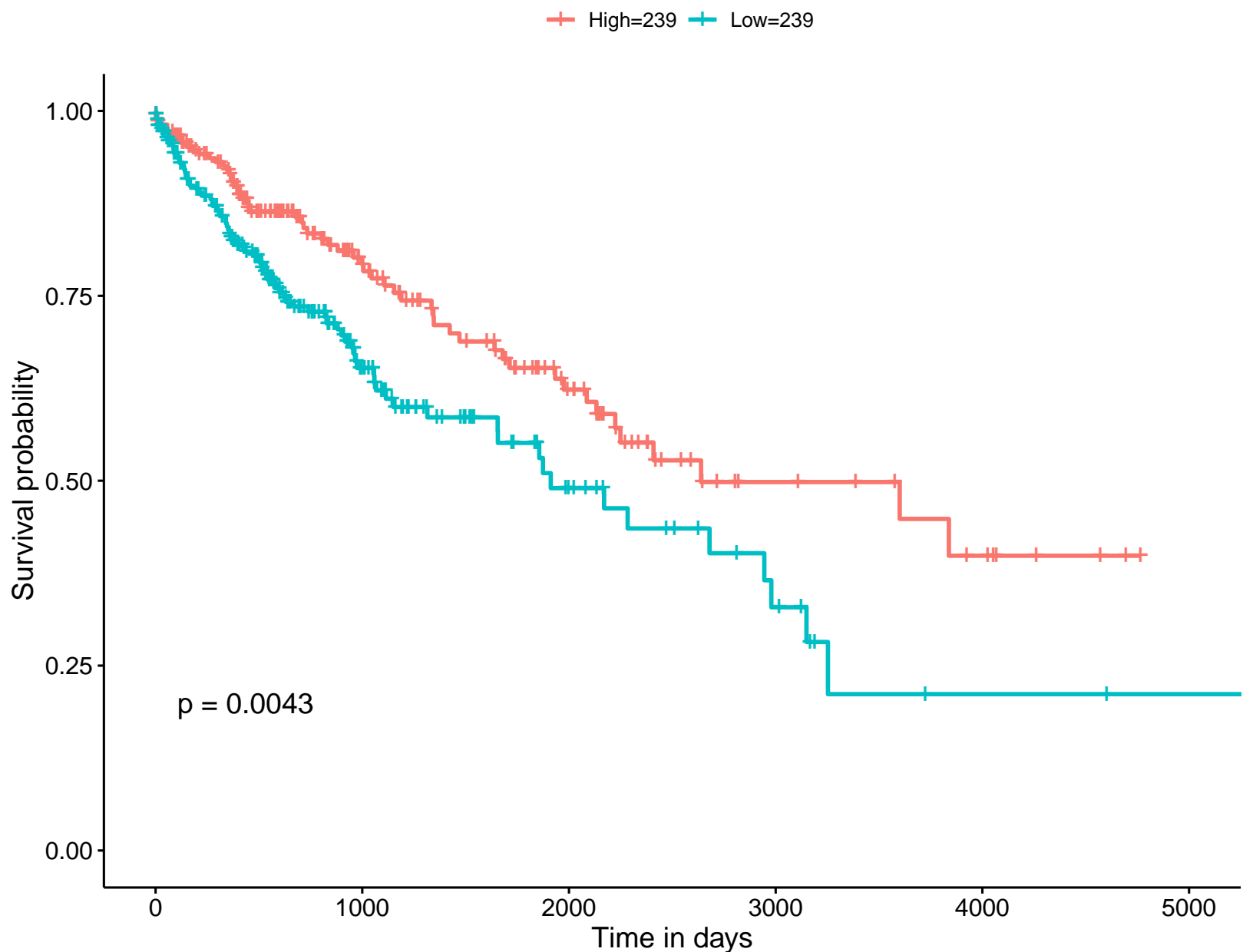

Supplement: Supplementary file 2 — Additional file 2: Overall Survival correlated with expression of miR−489−3p in TCGA−LUSC. [file 12931_2022_1988_MOESM2_ESM.pdf]
